# Full-length transcriptome sequencing reveals the molecular mechanism of potato seedlings responding to low-temperature

**DOI:** 10.1186/s12870-022-03461-8

**Published:** 2022-03-18

**Authors:** Chongchong Yan, Nan Zhang, Qianqian Wang, Yuying Fu, Hongyuan Zhao, Jiajia Wang, Gang Wu, Feng Wang, Xueyan Li, Huajun Liao

**Affiliations:** 1 Anhui Academy of Agricultural Sciences, Hefei, 230031 Anhui China; 2 Anhui Vocational College of City Management, Hefei, 231635 Anhui China; 3 Jieshou County Agricultural Technology Promotion Center, Jieshou, 236500 Anhui China; 4 Funan County Agricultural Technology Promotion Center, Funan, 236300 Anhui China

**Keywords:** potato, Second-generation sequencing technologies, Third-generation sequencing technologies, Full-length transcriptomes, Low-temperature stress

## Abstract

**Background:**

Potato (*Solanum tuberosum* L.) is one of the world's most important crops, the cultivated potato is frost-sensitive, and low-temperature severely influences potato production. However, the mechanism by which potato responds to low-temperature stress is unclear. In this research, we apply a combination of second-generation sequencing and third-generation sequencing technologies to sequence full-length transcriptomes in low-temperature-sensitive cultivars to identify the important genes and main pathways related to low-temperature resistance.

**Results:**

In this study, we obtained 41,016 high-quality transcripts, which included 15,189 putative new transcripts. Amongst them, we identified 11,665 open reading frames, 6085 simple sequence repeats out of the potato dataset. We used public available genomic contigs to analyze the gene features, simple sequence repeat, and alternative splicing event of 24,658 non-redundant transcript sequences, predicted the coding sequence and identified the alternative polyadenylation. We performed cluster analysis, GO, and KEGG functional analysis of 4518 genes that were differentially expressed between the different low-temperature treatments. We examined 36 transcription factor families and identified 542 transcription factors in the differentially expressed genes, and 64 transcription factors were found in the AP2 transcription factor family which was the most. We measured the malondialdehyde, soluble sugar, and proline contents and the expression genes changed associated with low temperature resistance in the low-temperature treated leaves. We also tentatively speculate that *StLPIN10369.5* and *StCDPK16* may play a central coordinating role in the response of potatoes to low temperature stress.

**Conclusions:**

Overall, this study provided the first large-scale full-length transcriptome sequencing of potato and will facilitate structure–function genetic and comparative genomics studies of this important crop.

**Supplementary Information:**

The online version contains supplementary material available at 10.1186/s12870-022-03461-8.

## Background

Potato cultivars (*Solanum tuberosum* L.) are sensitive to frost, low temperature is an important factor limiting potato geographical distribution and production, causing declines in potato yields in millions of hectares worldwide each year [1–3]. For potatoes, chemical or physical controls have an important role in controlling pests and diseases. However, these measures are not effective in preventing low-temperature frost damage. Therefore, genetic improvement of low-temperature tolerance will be the priority direction to solve this problem [4]. The previous studies have shown that, potato stops growing when the temperature is below 7 °C and will encounter chilling damage at -0.8 ℃, frost damage at -2 ℃, death at -3 ℃ [5]. At the same time, low temperature also has a series effects on the metabolism of malondialdehyde (MDA) [6], proline (Pro) [7], soluble sugar [8] and other substances [9], and lead to changes in molecular metabolism and subsequent physiological metabolism. However, the specific molecular mechanisms by which potato responds to low-temperature are not known and therefore research is urgently needed.

The acquisition of full-length cDNA sequences is the basis for structural and functional genomics studies. Firstly, it provides sequence information about expressed genes, which is important for functional analysis at the transcriptional and translational levels [10]. Secondly, it is useful for identifying coding regions in the genome, and facilitates the determination of the direction, order and boundaries of exons when predicting gene models [11]. Thirdly, this can help to analyze the different transcript isoforms produced by alternate splicing, which is an important method for increasing genetic and functional diversity in organisms. Finally, it helps to validate and correct the assembly of the genome, thus improving gene annotation [12]. Prior to this, it was necessary to use time-consuming, labor-intensive, and expensive Sanger sequencing to obtain full-length cDNA sequences [13, 14]. At present, the best way to generate transcriptome sequence is through second-generation sequencing technology (SGS)/ third-generation sequencing (TGS) hybrid sequencing [15, 16].

The second-generation DNA sequencing known as the second-generation sequencing technology is an epoch-making change to the first-generation sequencing technology. It revolutionized DNA sequencing, genome and transcriptome research [17]. In contrast to the first generation of Sanger sequencing, SGS technology has shown great progress in reducing costs, increasing yield and high sequence accuracy [18, 19]. During the past decades, SGS is widely used in genome and transcriptome research. However, the shortcomings of relatively short readings produced by SGS have not been resolved [20, 21]. In transcriptome sequencing projects, short reads are not conducive to bioinformatic analysis while reducing the accuracy of sequence assembly [22]. In recent years, the release of the TGS platform has solved this problem well [23]. TGS can produce reads up to 20 kb, albeit relatively low-quality [24]. The long read length of TGS is useful for the de novo genome and transcriptome assembly of higher organisms [25–27]. Its relatively high error rate has implications for sequence alignment and bioinformatics analyses, but can be improved and corrected by short, high-precision SGS reads [28, 29]. Therefore, hybrid sequencing approaches combining SGS and TGS technologies can provide high quality and more complete gene assembly in genome and transcriptome sequencing, which has been well illustrated in the previous studies [30, 31].

In the past few years, an increasing number of Pac-Bio full length transcriptomes have been sequenced and assembled. These studies have helped us to identify numerous new genes and alternatively spliced isoforms in many species, including human, Zebrafish, duck, broomcorn, Gossypium australe, sweet potato, and Nepenthes [14, 32–37]. On the whole, the above researches demonstrated that third-generation sequencing complements second-generation sequencing in the quantitative determination of eukaryotic transcripts and contributes to the discovery of an increasing number of alternatively spliced isomers [38].

Thus, we adopted joint TGS and SGS technology analysis to identify specific pathways and genes which involved in the different low temperature treatments of potatoes. We obtained 4518 differentially expressed genes (DEGs) from different low temperature times, including 542 TFs. Among them, *StLPIN10369.5, StCDPK16, StPME4759.3* and *StproC27072* genes showed significant differences in expression with low temperature treatment. We concluded that these differentially expressed genes may be related to resisting low-temperature stress in potatoes.

## Results

### Physiological response of potato to low-temperature stress

**Fig. 1:**
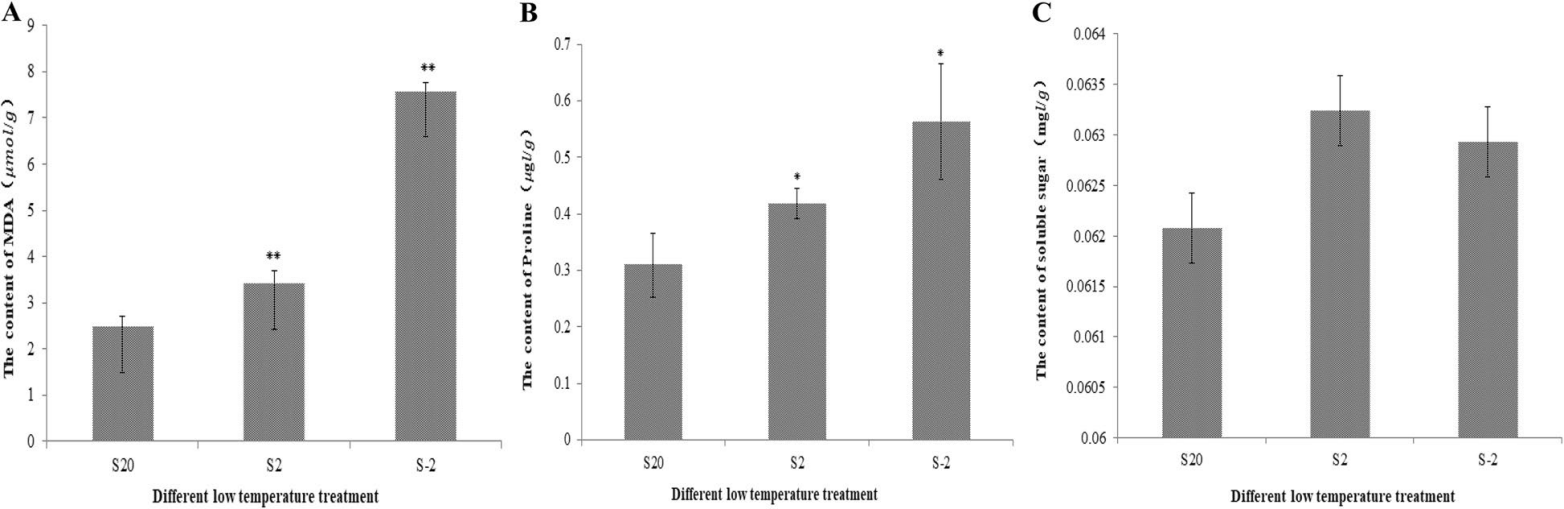
The effect of low-temperature stress on the physiological metabolism of potato. **A** is the effect of different low temperatures on MDA content in potato seedlings; **B** is the effect of low temperatures on proline content in potato seedlings; **C** is the effect of low temperatires on soluble sugar content in potato seedlings; S 20 is the control group which was treated at 20 °C for hours; S 2 is the treatment group which was treated at 2 °C for 4 h; S -2 is the treatment group which was treated at -2 °C for 4 h

In order to clarify the effects of low temperature stress on potato physiology metabolism, we measured MDA, soluble sugar and proline contents to observe the physiological changes in potato leaves exposed to 2 °C and -2 °C for 4 h (Fig. 1). As the temperature decreased, the MDA content increased gradually peaked at—2 °C and it is 3.12 times higher than the control group, (Fig. 1a). The proline content change trend was consistent with MDA; it was increased with the low temperature treatment and peaked at -2 °C (Fig. 1b). The soluble sugar content change trend was different from malondialdehyde and proline, it increased and then decreased and peaked at 2 °C (Fig. 1c). It can be seen that the MDA content in the potatoes increased continuously as the temperature decreased. The MDA content in the potatoes increased only 36.76% at the low-temperature treatment of 2 °C; when the temperature was reduced to -2 °C, the MDA content in the potatoes increased by 203.52%. In contrast, the change in soluble sugar content was not significant, and the content of SS also decreased at -2 °C. This may be due to the fact that when the temperature was lowered to -2 °C, serious damage was caused to the potato plant and the cell membrane had been damaged, resulting in a rapid increase in MDA content and inhibition of various enzyme activities, leading to a decrease in soluble sugar content, which is consistent with the results of Singh J. [39].

### Potato full-length transcriptome sequencing

**Fig. 2:**
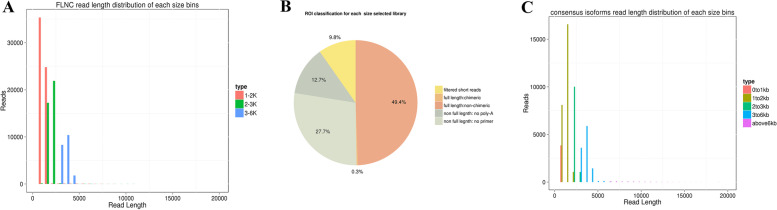
Summary of PacBio RS II single-molecule real-time (SMRT) sequencing. **A** is the number and length distributions of 220,035 reads in potato; **B** is the proportion of different types of PacBio reads in potato; **C** is the consensus isoforms read length distribution of each size bins

A total of 8.72 Gb of clean data was obtained from all four cells, with 8.72 billion nucleotides and 601,168 raw polymerase reads. A total of 3,990,120 subreads were obtained by filtering adaptor and low-quality sequences (Additional file 1: Table S1). High-quality reads of insertion (ROIs) were further generated from the circular consensus (CCS) after accurate filtering. The ROIs’ numbers from the library were 122,575 for 1–2 kb, 77,994 for 2–3 kb, and 44,918 for 3–6 kb, respectively (Additional file 1: Table S2). In total, 121,303 (49.75%) Full-length non-chimeric reads (FLNCs) were produced from ROIs, with average-lengths of 1293 bp, 2099 bp and 3464 bp in the corresponding libraries (Fig. 2a and b, Additional file 1: Table S3). However, the FLNC reads in each cDNA library contain duplicate isoforms. Based on ICE (Iterative isoform-clustering) analysis, the similarity sequences of FLNC reads were assigned to a cluster. And each cluster was identified as homozygous.Homozygous sequences were polished and integrated with non-full-length non-chimeric reads. After correction and classification by quiver and CEC (clustering for error correction) programs, 41,016 high-qualities (accuracy > 0.99) and 11,464 low-qualities polished isoforms were generated from the ROIs. The consensus isoform reads’ average lengths were modified to 909, 1506, 2294, 3535 and 9870 bp in 0–1 kb, 1–2 kb, 2–3 kb, 3–6 kb and > 6 kb libraries, respectively (Fig. 2c, Additional file 1: Table S4).

### PacBio ISO-seq results calibration

In order to further improve the accuracy of PacBio ISO-seq result, nine Illumina RNA-seq libraries constructed from potato leaves treated at different low temperatures were second generation sequenced to correct the polished isoforms of PacBio Iso-seq with Lordec software [40] and to quantify the full-length transcripts that had been obtained. Each RNA-seq sample produced more than 24,210,388 clean reads (Additional File 2, Table S5). Using RNA-seq short reads to correct the low-quality isoforms generated by ICE proofread, and then combined the corrected low-quality isoforms and high quality isoforms into the full-length isoforms. In ISO-seq analysis, multiple different isoforms may be generated from the same transcript, and these may also be assigned to different libraries, using TOFU software ( https://github.com/PacificBiosciences/cDNA_primer/wiki/What-is-pbtranscript-tofu%3F-Do-I-need-it%3F) to remove the redundant isoforms [41]. Finally, sequences with a coverage less than 0.85 and identity less than 0.9 were filtered, merged only the 5'-end exon with the difference in alignment, and obtained 24,658 non-redundant transcript sequences.

### Functional notes for new transcripts

**Fig. 3:**
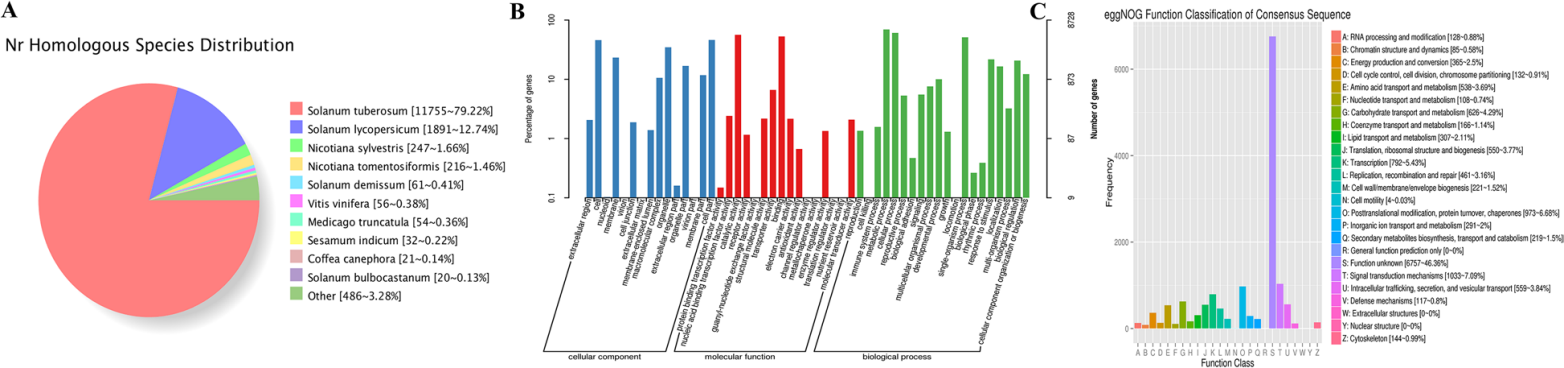
The Comparison of homologous species in Nr database

In order to further obtain comprehensive annotation information, all optimized transcripts were aligned to the nucleotide and protein databases, including the NCBI Non-Redundant Protein (NR), Gene Ontology (GO), Kyoto Encyclopedia of Genes and Genomes (KEGG), and Clusters of orthologous groups for eukaryotic complete genomes (KOG) with Blast software (version 2.2.26). A total of 14,864 transcripts were annotated; of these, 8,728 transcripts were annotated in the GO database and 6,570 transcripts were annotated in the KEGG database (Table 1). NR sequence alignment is used to predict the species most closely related to the potato. Through sequence alignment, 79.22% of the sequence is consistent with the published potato transcriptome sequence, and the second is the 12.74% sequence identity with tomato (Fig. 3).

**Table 1:** Annotated transcript number statistics table

**Anno**	COG	GO	KEGG	KOG	Pfam	Swissprot	eggNOG	nr	All
Annotated Number	6419	8728	6570	9625	11,992	10,671	14,251	14,846	14,86

Anno: Database for function annotation; Annotated Number: The number of new genes with corresponding database annotation information

### Alternative splicing (AS) event analysis

**Fig. 4:**
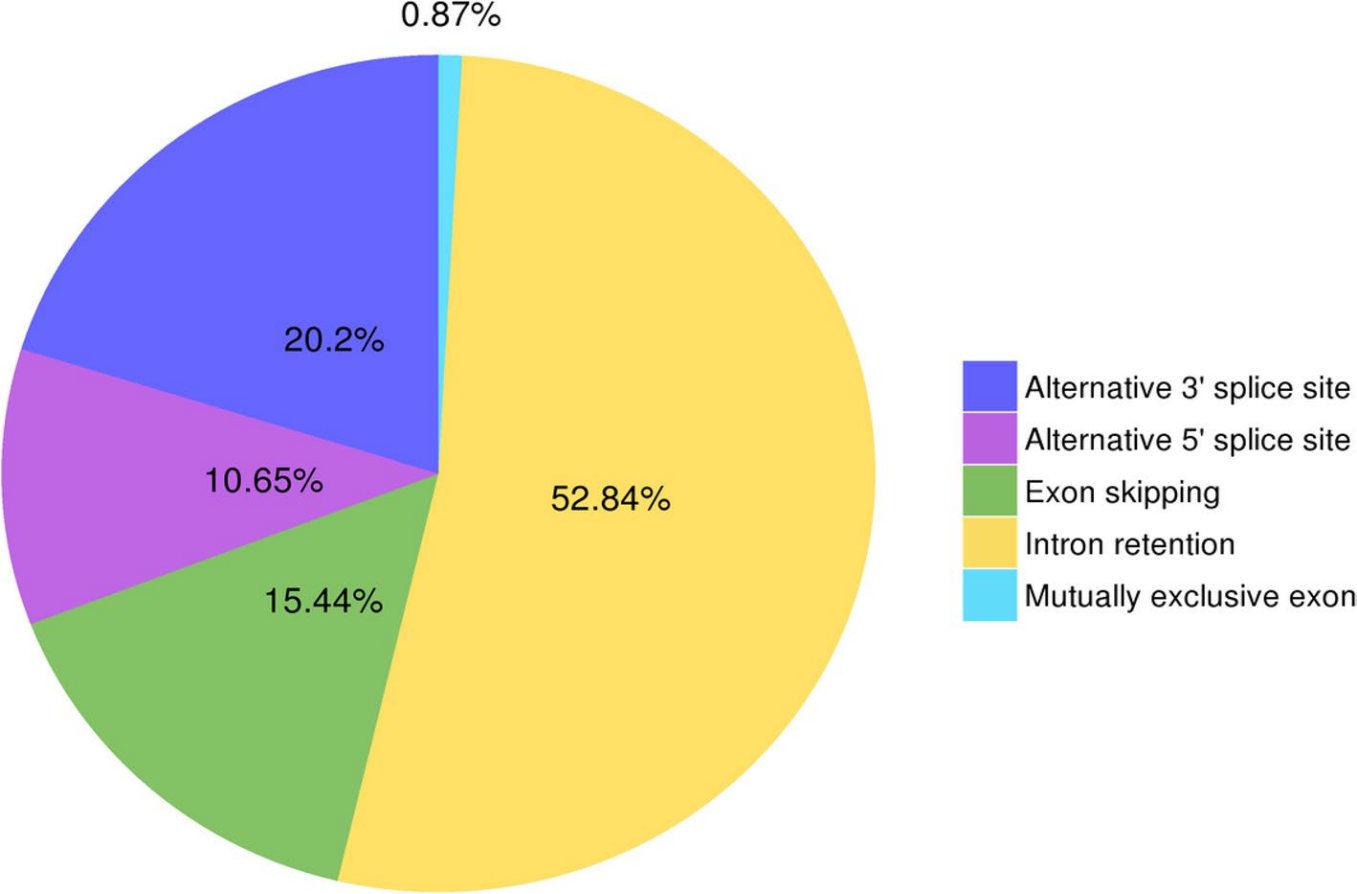
Statistics of the number of alternative splicing events. Alternative 3'splice site: alternative transcription termination site; Alternative 5'splice site: alternative transcription start site; Exon skipping: exon skipping; Intron retention: intron retention; Mutually exclusive exon: available Change exons

The precursor mRNA generated by gene transcription has multiple splicing methods, selects different exons, produces different mature mRNAs, and thus translates into different proteins, constituting a diversity of biological traits, this is alternative splicing. We analyzed alternative splicing events with Astalavista software (http://astalavista.sammeth.net/) and 6917 AS events were predicted in the sample. We found that intron retention had the highest proportion, reaching 52.84%; mutually exclusive exon had the lowest proportion, only 0.87% (Fig. 4).

### Simple repetitive sequence (SSR) analysis

**Fig. 5:**
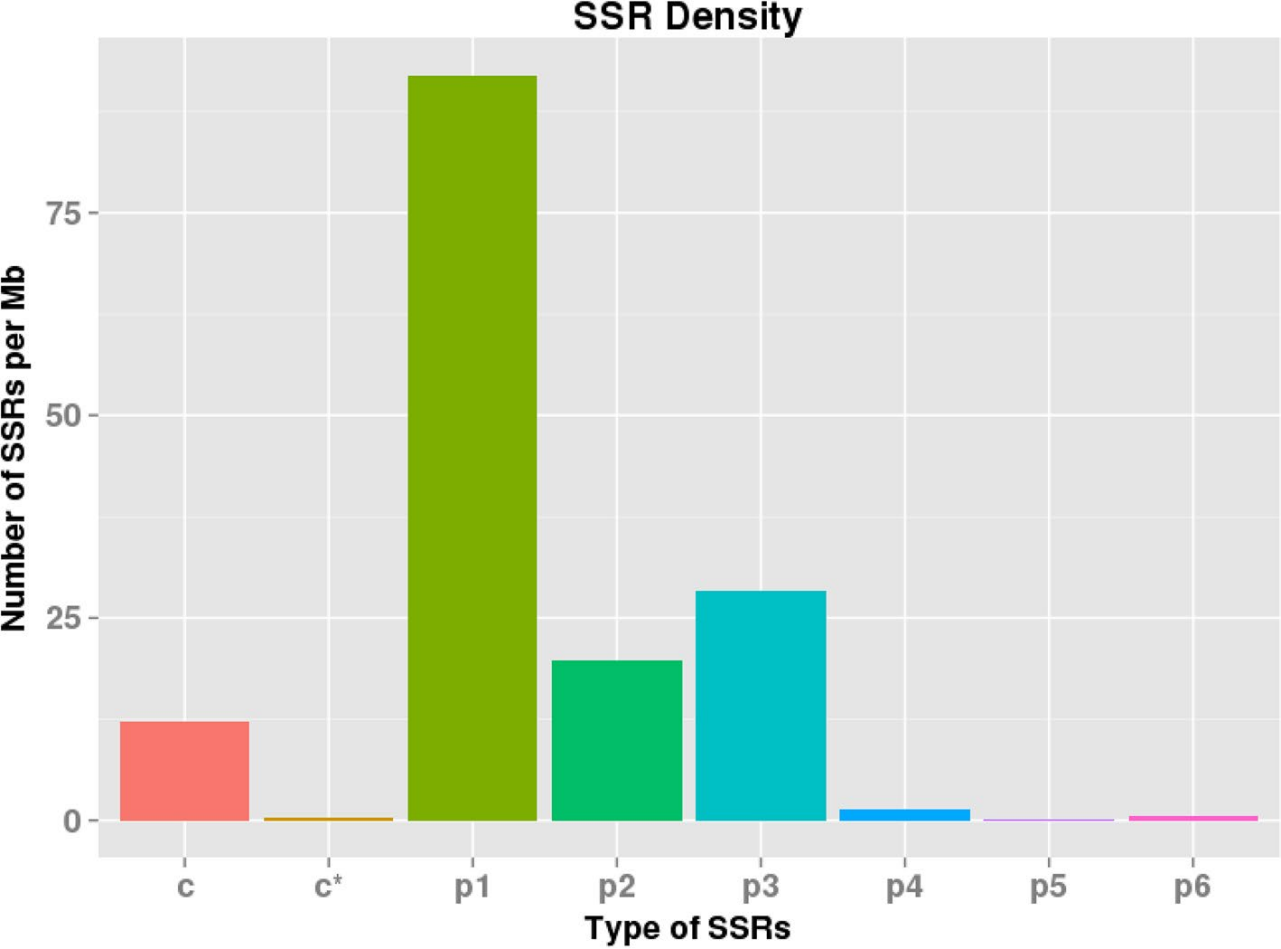
The distribution of different type of SSRs

We analyzed SSR on transcripts over 500 bp selected from new transcripts with MIcroSAtellite identification toolsoftware which could identify 7 types of SSR, they are Mono nucleotide (p1), Di nucleotide (p2), Tri nucleotide (p3), Tetra nucleotide (p4), Penta nucleotide (p5), Hexa nucleotide (p6), compound SSR (c) and compound SSR with overlapping positions (c*) [42]. It can be seen that there are significant differences in the number and density distribution of different types of SSR (Table 2, Fig. 5), p1 had the largest number and distribution density, followed by p3, and p5 the least.

**Table 2:** The number and density distribution of SSR

type	c	c*	p1	p2	p3	p4	p5	p6	Total
number	437	14	3310	711	1020	47	6	18	5563
density	12.15106	0.389279	92.03662	19.7698	28.36174	1.306864	0.166834	0.500501	

c: indicated compound SSR, c* indicated compound SSR with overlapping positions

### Coding sequence (CDS) prediction

**Fig. 6:**
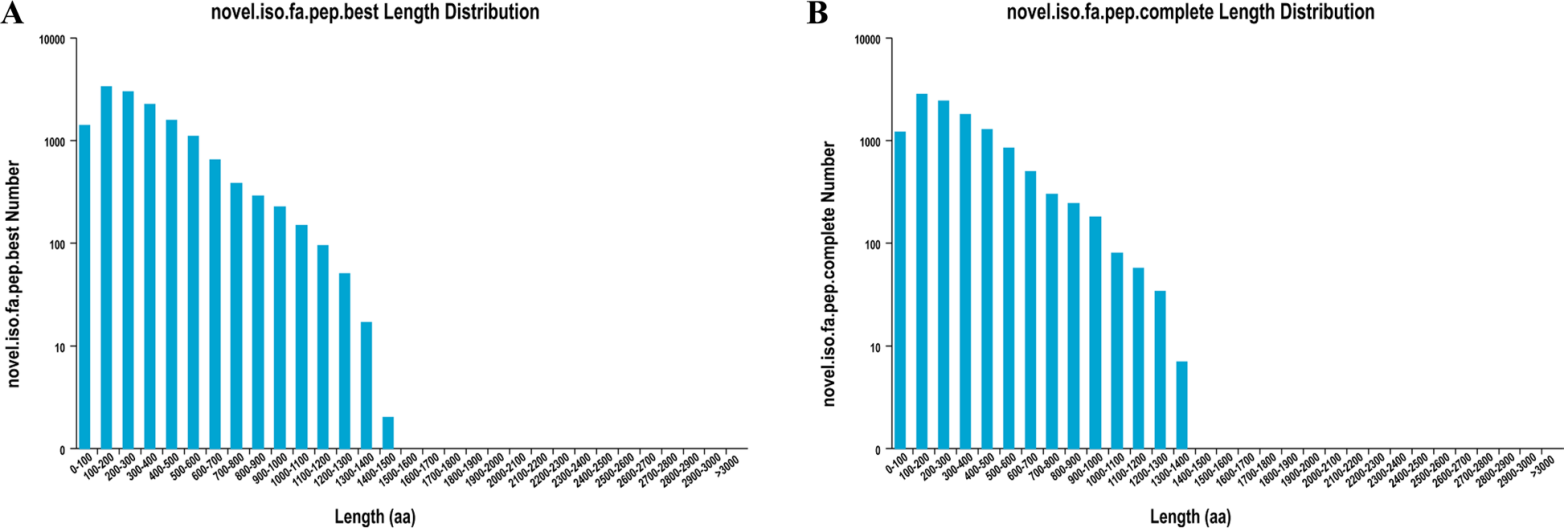
Predicted CDS-encoded protein length distribution map. **A** is the predicted best CDS-encoded protein length distribution map; **B** is the predicted complete CDS-encoded protein length distribution map

All new transcripts were predicted for protein sequence and CDS using TransDecoder software (v3.0.0), which can be identified reliable potential coding region sequences from transcript sequences. We obtained 14,435 ORFs, including 11,665 (80.81%) complete ORFs. From Fig. 6, the predicted length of the protein sequence encoded by the ORFs region and the protein sequence encoded by the complete ORFs region have the same change trend of length distribution, the maximum is 100–200 amino acids, and the second is 200–300, the sequence is gradually reduced.

### Alternative polyadenylation (APA) Identification

**Fig. 7:**
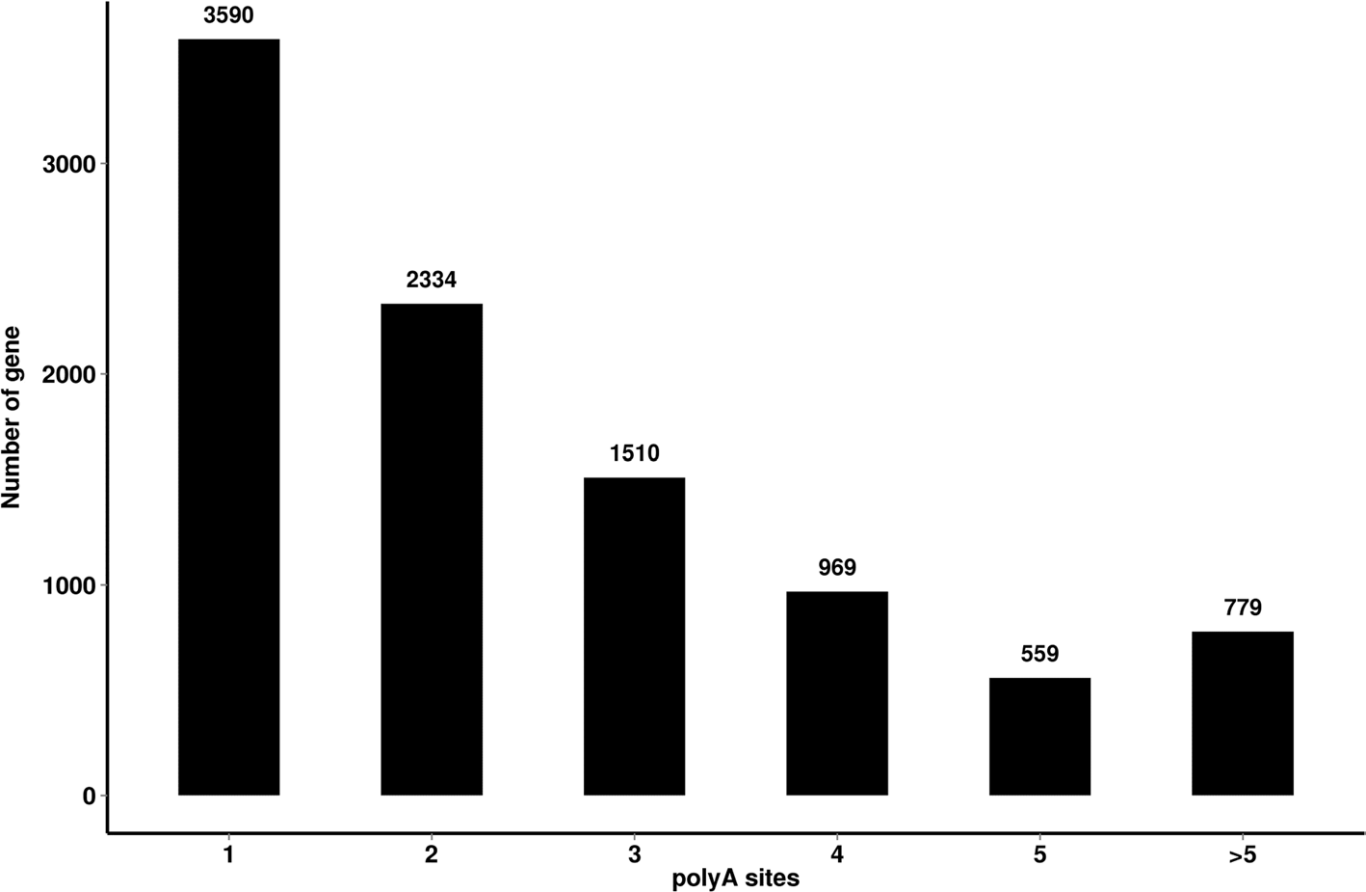
Distributions of polyadenylation sites in potato gene. Abscissa: the number of polyadenylation sites; Ordinate: the number of genes

Most genes in eukaryotes can produce a variety of different mRNA 3' ends via APA [35]. It has been found that poly (A) sites are dynamically regulated during tissue development and external environmental stimuli [43]. The TAPIS pipeline was used to analyze the heterogeneity and genomic poly (A) sites formed at the 3' ends of all potato transcripts. 159,194 reads were detected in 9741 genes with 25,294 poly(A) sites. And 3590 genes (36.86%) have single poly (A) sites in these genes (Fig. 7).

### Analysis of differentially expressed genes in response to low-temperature stress

**Fig. 8:**
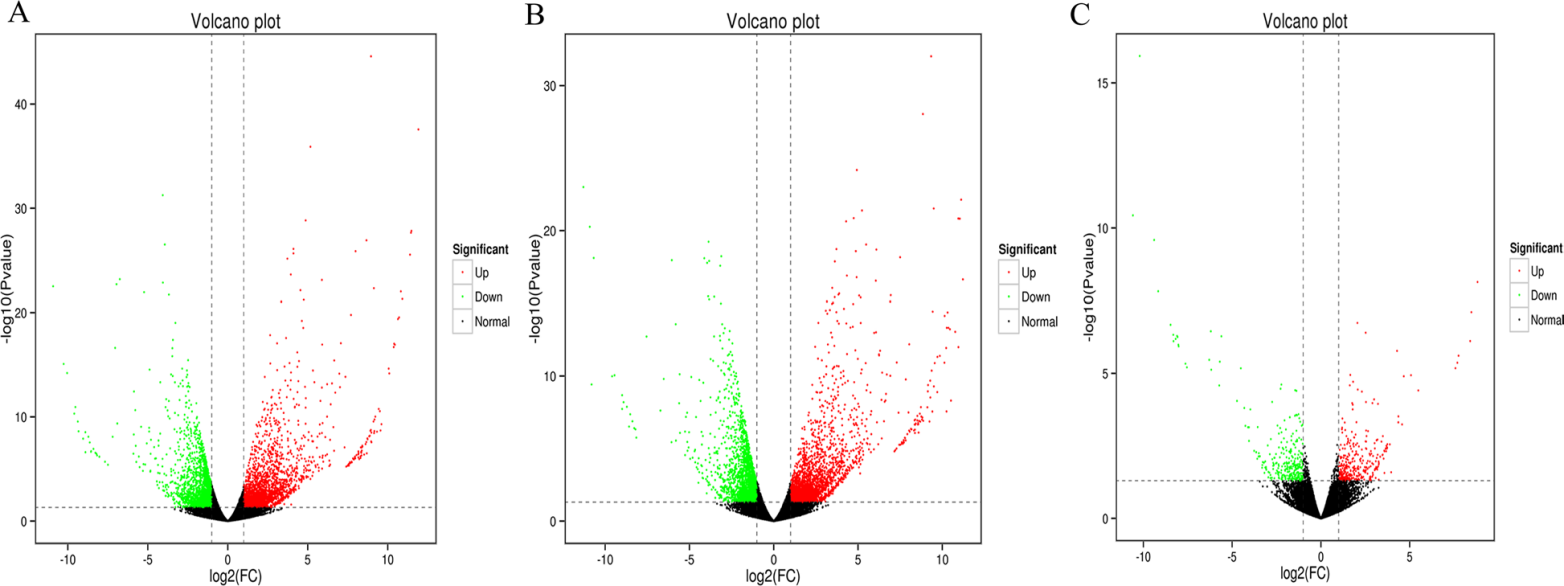
Differentially expressed genes volcano map. Each point in the volcano map represents a gene. The abscissa represents the logarithm of the difference in expression of a certain gene in two samples; the ordinate represents the negative logarithm of the change in gene expression. The larger the absolute value of the abscissa, the greater the difference in expression between two samples; the larger the ordinate value, the more significant the difference and the more reliable the differentially expressed genes. In the figure, the green dots represent down-regulated differentially expressed genes, the red dots represent up- regulated differentially expressed genes, and the black dots represents non-differentially expressed genes. **A** is the differentially expressed transcript volcano map of S20 vs S2; **B** is the differentially expressed transcript volcano map of S20 vs S-2; **C** is the differentially expressed transcript volcano map of S2 vs S-2

To identify differentially expressed genes (DEGs), we used DESeq (http://www.bioconductor.org/packages/release/bioc/html/DESeq.html) to analyze gene expression differences between control and low-temperature treated groups. The filtered clean reads from Illumina RNA-seq were mapped to the reference genome. Of all the reads, 78.70–82.85% were uniquely mapped reads, 4.87–7.13% were multiple aligned reads, and 0.07–0.42% were too many multiple aligned reads (Table 3). A total of 4518 DEGs were obtained between S20 and S2, 2486 DEGs were significantly up-regulated, 2032 DEGs were significantly down-regulated; 4498 DEGs were obtained between S20 and S-2, 2369 DEGs were significantly up-regulated, 2129 DEGs were significantly down-regulated; 654 DEGs were obtained between S2 and S-2, 342 DEGs were significantly up-regulated, 312 DEGs were significantly down-regulated (Table 4, Fig. 8).

**Table 3:** Statistics of comparison between transcriptome sequencing data and reference genome sequence

Sample		Total Reads	Uniquely mapped reads %	% of reads mapped to multiple loci	% of reads mapped to too many loci
S20	T01	28,270,361	81.05%	5.52%	0.08%
	T02	27,318,299	82.24%	5.87%	0.07%
	T03	32,928,931	81.98%	5.81%	0.09%
S2	T10	24,525,892	80.98%	6.62%	0.32%
	T11	24,275,418	78.70%	7.13%	0.42%
	T12	26,140,364	82.85%	4.89%	0.05%
S-2	T13	24,759,490	82.24%	5.33%	0.12%
	T14	24,210,388	81.82%	4.87%	0.07%
	T15	25,615,720	79.14%	6.10%	0.20%

The Reads that mapped to more than 10 loci were counted as mapping to too many loci

**Table 4:** Low-temperature treatment differentially expressed gene statistics

DEG set	DEG Number	up-regulated	down-regulated
S20 vs S2	4518	2486	2032
S20 vs S-2	4498	2369	2129
S2 vs S-2	654	342	312

**Fig. 9:**
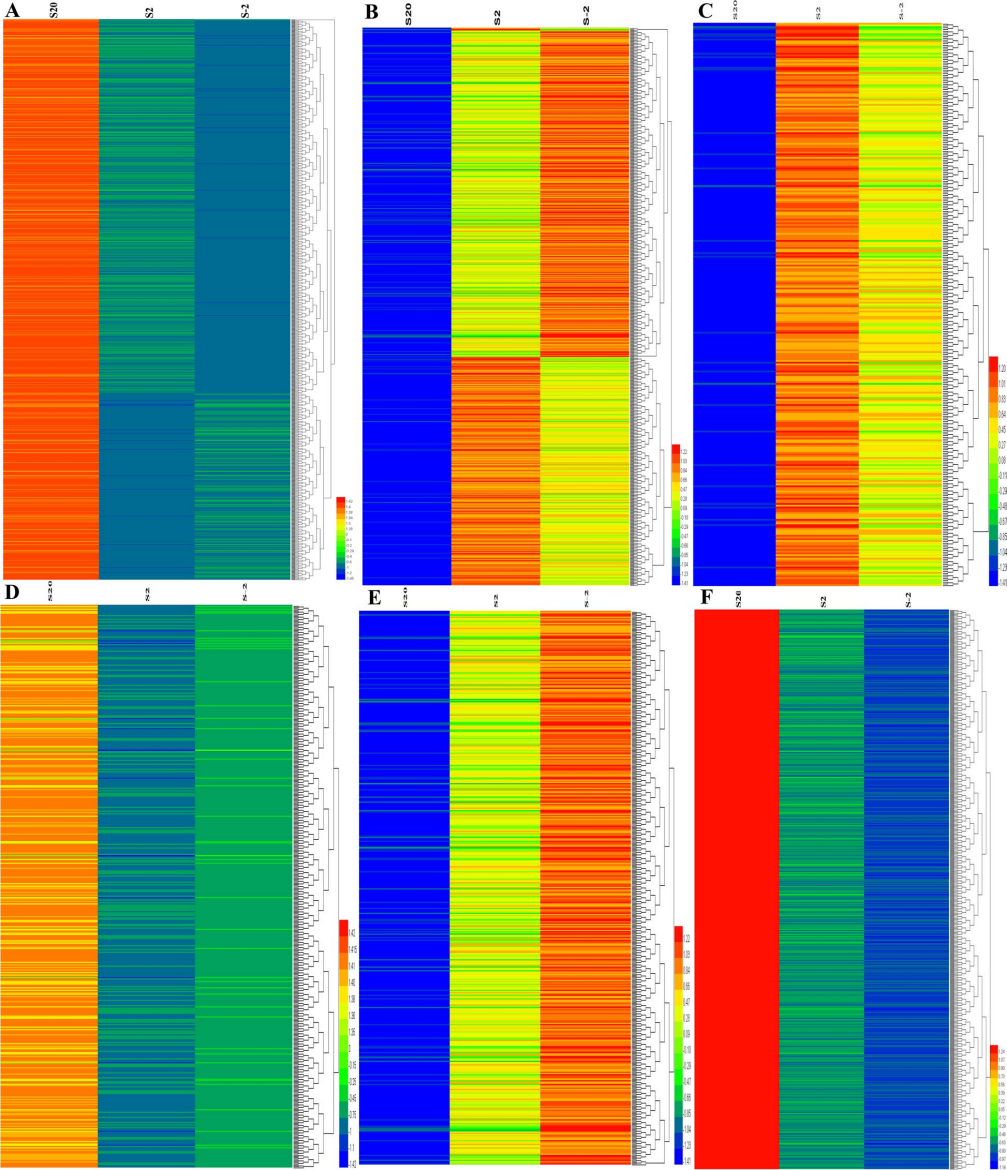
Heat map of DEGs during low-temperature stress in the potato. The color represents gene expression values (the red corresponds to genes with high expression and the blue corresponds to gene with low expression). S20, S2 and S-2 correspond to the libraries obtained in the temperature 20 °C, 2 °C and -2 °C respectively. **A** up-regulation at 20 °C but down-regulation at other 2 temperatures; **B**: down-regulation at 20 °C but up-regulation at other 2 temperatures; **C**: up-regulation at 2 °C but down-regulation at other 2 temperatures; **D**: down-regulation at 2 °C but up-regulation at other 2 temperatures; **E**: up-regulation at 2 °C but down-regulation at other 2 temperatures; **F**: up-regulation at -2 °C but down-regulation at other 2 temperatures

Cluster analysis of DEGs was conducted and the results showed in Fig. 9 and Additional file 3. All the DEGs were assigned to four main clades: 1. the DEGs showed down-regulated at 20 °C and up-regulated during low-temperature stress (2 °C and -2 °C),; 2. The DEGs showed up-regulated at 20 °C, but most showed a downward trend during low-temperature stress (2 °C and -2 °C); 3. The DEGs showed up-regulated at the low temperature (2 °C and -2 °C), but lower at control temperatures (20 °C); In the last clade, the DEGs showed down-regulated at the low-temperature (2 °C and -2 °C), but higher at control temperature (20 °C).

### Functional annotation of the differentially expressed genes

**Fig. 10:**
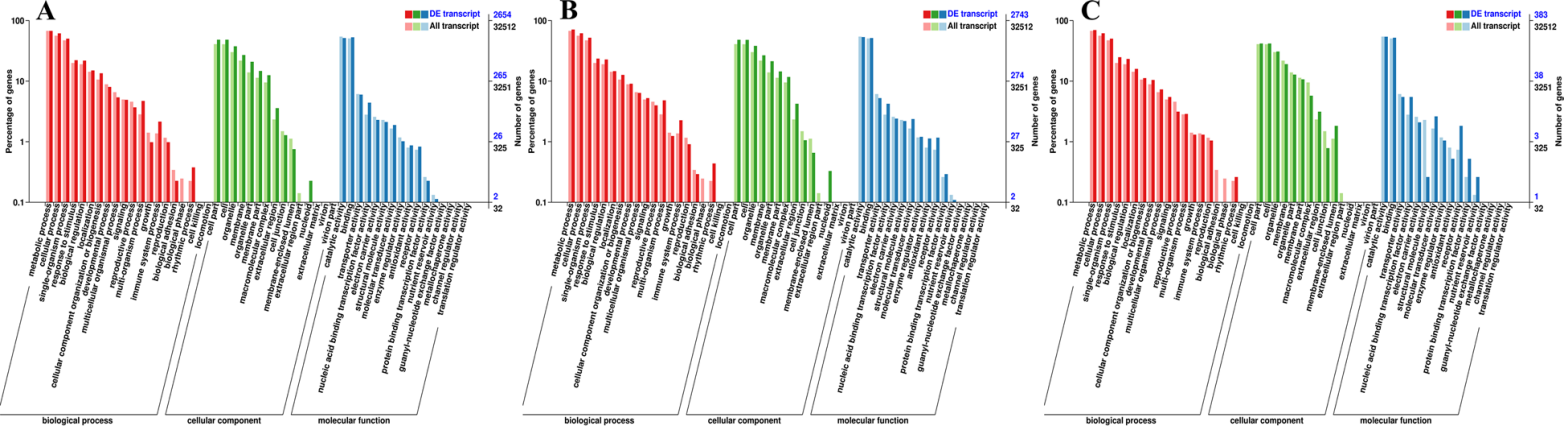
GO enrichment analysis mapped to potato during low-temperature stress in the potato. A is the GO enrichment analysis between S20 vs S2; B is the GO enrichment analysis between S20 vs S-2; C is the GO enrichment analysis between S2 vs S-2

GO enrichment was applied on the DEGs with different low-temperature treatments. 1053 significantly enriched GO terms have been identified in S20 vs S2 (KS < 0.05). There are 622, 82, and 349 significantly enriched GO terms in biological process, cellular component, and molecular function, respectively; 1032 significantly enriched GO terms have been identified in S20 vs S-2 (KS < 0.05). There are 359, 77, and 596 significantly enriched GO terms in molecular function, cellular component, and biological process, respectively; 1049 significantly enriched GO terms have been identified in S2 vs S-2 (KS < 0.05). There are 625, 79, and 345 significantly enriched GO terms in biological process, cellular component, and molecular function, respectively (Additional file 4). Moreover, with low-temperature treatment, the biological processes have the highest proportion (57.75 ~ 59.58%), and the cellular component has the lowest proportion (7.46 ~ 7.79%) (Fig. 10).

**Fig. 11:**
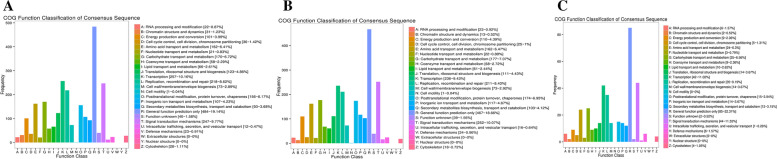
COG annotation classification statistics of differentially expressed genes. The abscissa is the content of each COG classification, and the ordinate is the number of genes; **A** is the COG annotation classification statistics analysis between S20 vs S2; **B** is the COG annotation classification statistics between S20 vs S-2; **C** is the COG annotation classification statistics analysis between S2 vs S-2

The COG is a database for homologous classification of gene products. It is often used to identify direct homologous genes by comparing protein sequences from many species. DEGs were the most enriched in function R (General function prediction only), followed by function K (transcription) and function T (Signal transduction mechanisms) in S20 vs S2; DEGs were the most enriched in function R (General function prediction only), followed by function K (transcription) and function T (Signal transduction mechanisms) in S20 vs S-2; DEGs were the most enriched in function R (General function prediction only), followed by function T (transcription) and function K (Signal transduction mechanisms) in S2 vs S-2 (Fig. 11, Additional file 5).

**Fig. 12:**
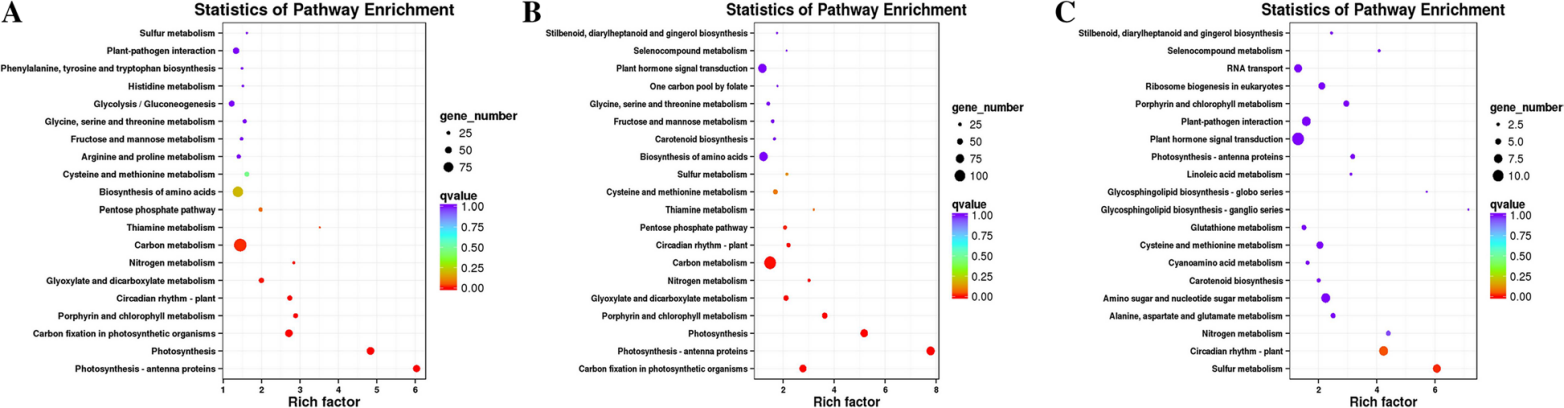
Scatter plot of enrichment of differentially expressed genes in the KEGG pathway. Each circle in the figure represents a KEGG pathway, the ordinate indicated the name of the pathway, and the abscissa is the enrichment factor, which represents the ratio of the proportion of genes annotated to a pathway in the differential gene to the proportion of genes annotated to the pathway in all genes. The larger the enrichment factor the more significant the enrichment level of differentially expressed genes in this pathway. The color of the circle represents the q-value, which is the *P*-value after multiple hypothesis testing corrections. The smaller the q-value, the more reliable the significance of the enrichment of differentially expressed genes in the pathway; the size of the circle indicates the number of enriched genes in the pathway, the larger the circle, the more genes. **A** is the KEGG enrichment analysis between S20 vs S2; **B** is the KEGG enrichment analysis between S20 vs S-2; **C** is the KEGG enrichment analysis between S2 vs S-2

The DEGs were also subject to KEGG analysis, 1747, 1768, and 246 DEGs were assigned to 117, 113, and 73 pathways in S20 vs S2, S20 vs S-2, and S2 vs S-2, respectively (Additional file 6). The DEGs significantly enriched in the top 20 significant KOs are shown in Fig. 12. The highly enriched pathways of DEGs were Photosynthesis-antenna proteins (ko00196), Photosynthesis (ko00195), and Carbon fixation in photosynthetic organisms (ko00910) in S20 vs S2. Carbon fixation in photosynthetic organisms (ko00710), Photosynthesis—antenna proteins (ko00196), and Photosynthesis (ko00195) were the highly enriched pathways in S20 vs S-2. The highly enriched pathways of DEGs were Sulfur metabolism (ko00920), Circadian rhythm-plant (ko04712), and Nitrogen metabolism (ko00910) in S2 vs S-2. The highly enriched pathways of DEGs were Photosynthesis—antenna proteins (ko00196), Porphyrin and chlorophyll metabolism (ko00860), Circadian rhythm-plant (ko04712), and Glyoxylate and dicarboxylate metabolism (Ko00260) in S20 vs S2 vs S-2.

### Analysis of transcription factors in response to low temperature stress

Transcription factors (TFs) are key components involved in transcriptional regulatory systems in organisms, particularly in the context of abiotic stresses, and as a result, they have become a hot topic of research on various topics. In order to highlight the TFs available in our transcript database, we obtained 542 putative TF members from our transcript database by comparing 36 TF gene families (Additional file 7). When comparing the number of TFs we predicted with the number of known TFs that have been published, we found that in most TF gene families, the number of TFs we predicted is underrepresented, and the SAP family with the highest proportion is only 12.2% (Additional file 8). This is normal, as the expression of most TFs is spatiotemporally controlled, they are difficult to detect simultaneously in a transcriptional level.

Through analysis, we have found more than 10 TFs in each of 20 TF families; among them, there are 64 TFs in AP2 families with the most TFs; followed by the C3H TF family, with 61; MYB TF family has 51; CPP, WOX, HD-ZIP and NY-FC TF families found the least number of TFs, only one. Among these TFs, most are related to plant resistance, growth and morphogenesis, which are consistent with the functions of TFs and previous studies [44–46].The most significant difference in the expression level of TFs was PGSC0003DMT400056121, whose expression in S-2 is 2^10.5^ times that of S20, followed by PB.5665.2, whose expression in S2 is 2^6.65^ times that of S20. At the same time, some TFs had a significant decrease in their expression with low temperature stress, indicating that their expression was inhibited by low temperature stress. For example, the expression level of PGSC0003DMT400024670 in S20 is 2^4.42^ times that of S-2, and the expression level of PGSC0003DMT400000901 in control S20 is 2^4.33^ times and 2^4.28^ times higher than that in S2 and S-2 treated with low-temperature, respectively.

### qRT-PCR validation of some differentially expressed genes

**Fig. 13:**
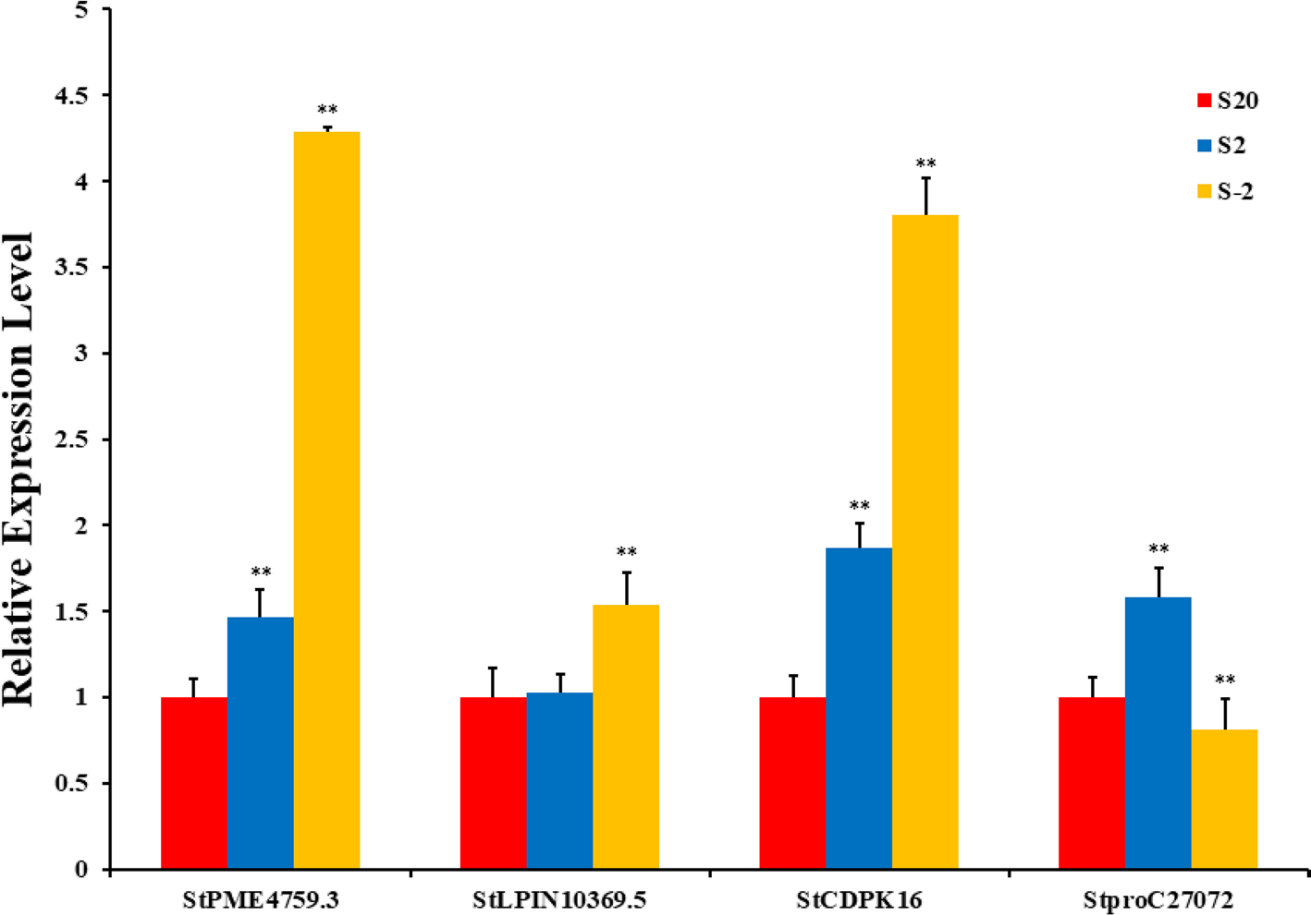
The qPCR validation of partial genes during low-temperature stress in the potato. Note: *P < 0.05; **P < 0.001; the number of biological replicates = 3

We verified the expression patterns of 4 genes (pectinesterase, phosphatidate phosphatase, calcium-dependent protein kinase, and pyrroline-5-carboxylic acid reductase) in different low temperature conditions using qRT-PCR.The results were showed in Fig. 13 and Additional file 9, it can be seen that the expression of pectinesterase (*StPME4759.3*) showed a gradual increase as the treatment temperature decreased and reached the maximum at -2 °C. The expression level of phosphatidate phosphatase (*StLPIN10369.5*) increased significantly when the temperature dropped to -2 °C, and it mainly catalyzes the formation of diacyl-sn-glycerol 3-phosphate into diacyl-sn-glycerol, which finally forms triglycerides. The increase in triglyceride content helps to increase the content of unsaturated fatty acids in plants, thereby improving the plant's ability to resist low temperature stress. The expression level of calcium-dependent protein kinase (*StCDPK16*) was consistent with the first two genes, which mainly promoted the flow of calcium-ions from extracellular to intracellular. The expression of *StCDPK16* was consistent with the first two genes, which mainly promoted the flow of calcium ions from extracellular to intracellular, thus promoting signal transduction and improving the ability of plants to respond to low temperature stress. The expression of pyrroline-5-carboxylic acid reductase (*StproC27072*) increased at 2 °C, whereas decreased at -2 °C, which mainly catalyzes the formation of L-proline from L-1-pyrroline-5-carboxylic acid.

## Discussion

SGS has the advantages of low cost, high accuracy and short sequencing time, but shorter sequence read lengths; TGS has longer sequence reads but higher error rates [47]. As a result, more and more studies on the response to plant stress are now beginning to make use of combined SGS and TGS sequencing methods, which can provide a more complete and high-quality assembly at the transcriptome level [48–50]. In our study, we investigated the transcriptome assembly of potato plant leaves under low temperature stress using a combined SGS and TGS approach, and identified key functional and regulatory genes involved in low temperature stress. We obtained 24,658 non-redundant sequences with an average ROI of sufficient length to represent full-length transcripts.

When plants were subjected to adversity, they would make a series of emergency responses. AS played an important role. Adversity stress derived AS to significantly affect the expression of genes related to stress response pathways [51, 52]. Under low-temperature stress, the AS of most expressed genes in Arabidopsis changed, and AS was very important for plants to resist temperature stress [53]. Under salt stress, the significantly differently alternative splicing genes (DASGs) in Arabidopsis respond to stress in a relatively independent manner from DEGs [51]. It can be seen that AS played a key role in the response of plants to various biotic and abiotic stresses. In this study, 6917 AS were predicted, indicating that when potatoes were under low-temperature stress, AS significantly affected the expression of genes related to stress response pathways.

SSR is a type of tandem repeat sequence consisting of 1–6 nucleotides as repeating units of dozens of nucleotides. These sequences are conservative, but there will be differences between individuals, which results in site polymorphism can be used in molecular marker-assisted breeding, which is of great significance to the study of plant resistance genetic breeding. At present, SSR markers have been well applied in molecular genetic testing of different resistant plants such as apples [54]. Sun et al. used SSR markers to identify 4 resistance sites related to phytophthora sojae resistance through association analysis [55]. A. K. Singh et al. used 125 SSR markers to perform an association analysis on 91 rice varieties for disease resistance, and screened 32 SSR loci associated with resistance to rice blast and 19 associated with resistance to sheath blight [56]. A total of 5563 SSRs were obtained in this study, which provided abundant molecular markers for the later development of potato cold resistance genetic breeding.

Polyadenylation is an important process in eukaryotic post-transcriptional processing that serves a signal recognition function, prevents mRNA degradation and participates in the protein translation process [57]. The selective use of polyadenylation sites by genes for polyadenylation is known as alternative Poly adenylation (APA). With the development of high-throughput sequencing, APA had been found to be widely present in plants and animals [58, 59]. It was found that 70% of the genes in Arabidopsis have multiple poly (A) loci [60]. Among them, *AtCPSF30* and *AtCPSF100* are important for their disease resistance, and their mutants show sensitivity to bacterial infestation [61]. APA plays a important role in plant response to the environment [62]. A total of 9741 APAs were obtained in this study, indicating that APAs play an important role in the response of potato to low-temperature stress.

It has been shown that the physiological changes in plants in response to low temperature stress are complex. Under low temperature stress, plant leaves show more significant changes in cell membrane structure and osmoregulation. For example, in *Medicago falcata* and eggplant, which received low temperature stress, their MDA, proline and soluble sugar levels increased and electrolyte leakage occurred [63, 64]. In low-temperature treated transgenic potatoes, plants accumulated higher amounts of MDA, sucrose and proline and showed higher activity of pectin esterase, pyrroline-5-carboxylate reductase and phosphatidate phosphatase [65]. In this study, an increase in soluble sugar, proline, and MDA contents was observed in the leaves of low temperature treated potato plants, it is suggested that these osmotic regulators may play a role in increasing the stability of plant cell membranes and protecting them during dehydration of potatoes under low temperature stress, while balancing the osmotic pressure inside and outside the cells. Thus, the changes in proline and soluble sugar may play a key role in the osmotic regulation by low-temperature in potato leaf, which is consistent with the study of Wan Y-Y et al. [66].

In this paper, a total of 6381 DEGs were identified as responsive to low temperature stress, of which 4518 DEGs were between S20 and S2, 4498 DEGs were between S20 and S-2, 654 DEGs were between S2 and S-2.

The most significant difference in up-regulated expression between S20 and S2 was triosephosphate isomerase, with a 2^9.15^-fold up-regulation, followed by glycerol-3-phosphate transporter with a 2^5.57^-fold up-regulation; the most significant down-regulation was E3 ubiquitin-protein ligase, with a 2^3.79^-fold decrease. The most significant difference in up-regulated expression between S20 and S-2 was in serine/arginine-rich splicing factor, which was up-regulated by 2^11.12^-fold, followed by protochlorophyllide reductase, which was up-regulated by 2^10.37^-fold; the most significant down-regulation was ubiquitin conjugation factor, with a 2^10.81^-fold decrease in expression. The most significant difference in up-regulated expression between S2 and S-2 was triosephosphate isomerase, with a 2^8.47^-fold up-regulation, followed by d-3-phosphoglycerate dehydrogenase, with a 2^7.75^-fold up-regulation; the most significant down-regulation was RNA polymerase sigma factor sigF, with a 2^10.59^-fold decrease in expression. It can be seen that the expression of triosephosphate isomerase increased significantly with low temperature treatment, which may be related to its catalytic production of glyceraldehyde 3-phosphate, thereby increasing plant stress tolerance [67].

All DEGs were grouped into four sub-clusters, and then performed a KEGG enrichment analysis. The most enriched pathway between S20 and S2 was “photosynthesis-antenna proteins”, followed by “photosynthesis”. This indicated that the low-temperature stress significantly affected photosynthesis in the leaves of potatoes [68]. “Carbon fixation in photosynthetic organisms” was the most enriched pathway between S20 and S-2, this showed that carbohydrate metabolism was very important for potatoes to adapt to low temperature [69, 70]. The highly enriched pathway between S2 and S-2 was “sulfur metabolism”. Sulfur is a key component of plant cysteine, which is used by plants as a precursor to synthesize numerous metabolites with important biological functions that are directly related to plant resistance to stress [71]. Sulfur is also an important component of proteins and biofilms, and the interconversion of -sh groups and s–s- in proteins constitutes the redox system in plants. Therefore, sulfur metabolism is of great importance to plants in response to low-temperature stress [72]. The common KEGG pathways between the different low temperature treatments were “Photosynthesis—antenna proteins” “Porphyrin and chlorophyll metabolism” “Circadian rhythm-plant” “Glyoxylate and dicarboxylate metabolism” “Nitrogen metabolism” and “Cysteine and methionine metabolism”. These KEGG pathways are mainly involved in metabolic synthesis, photosynthesis, and circadian rhythms, indicating that these metabolic pathways are deeply involved in the response of plants to low-temperature stress.

TFs refer to a class of proteins that can bind to DNA in a sequence-specific manner and regulate transcription [73]. Under adversity conditions, plants stimulate TFs through a series of signal transmissions, which combine with corresponding cis-acting elements to activate the RNA polymerase II transcription complex, thereby initiating the expression of specific genes and responding to external signals [74]. Related TFs can regulate the functional genes expression under stress conditions, and their overexpression will activate or inhibit the expression of many stress resistant genes, thereby changing the stress resistance of plants [75].

Many TFs, including MYB, bHLH, NAC, bZIP, C2H2, ERF, and WRKY types, have been demonstrated by transcriptomic and other approaches to be involved in plants response to low-temperature stress [76, 77]. In our study, 542 TFs were differentially expressed between low temperature treatments, and they were grouped into 36 TF gene families. The most abundant TF family was the AP2 family, followed by the C3H, MYB, CPP, WOX, HD-ZIP, and NY-FC families, and the dynamic changes in the expression of genes associated with these TFs may indicate their important function in low temperature stress in potato. These results are the same with the previous studies on TFs which involved in plants response to low-temperature stress and suggest that members of the AP2 family play a critical role in potato low-temperature response [78, 79]. At the same time, the most significant difference in the expression level of TF is PGSC0003DMT400056121, whose expression in S-2 is 2^10.5^ times that of S20, followed by PB.5665.2, whose expression in S2 is 2^6.65^ times that of S20. However, there were also some TFs have a significant decrease in their expression with low-temperature stress, suggesting that their expression is inhibited by low-temperature stress. This indicated that while low temperature stress promoted the expression of cold tolerance-related TFs, it also inhibited the expression of some TFs unrelated to plant resistance, thereby maximizing the ability of plants to resist low temperature stress, which is consistent with the results of Sun X. et al. [80].

Plants have developed many metabolic pathways and mechanisms to help them minimize the damage caused by low temperatures. Through the analysis of low-temperature stress response genes, we found that *StPME4759.3*, *StLPIN10369.5*, *StCDPK16*, *StproC27072* were involved in potato response to low temperature stress.

Pectin is an important component of plant cell wall and plays an important role in maintaining cell wall stability and resistance to adversity, accounting for 35% of the cell wall components of dicotyledons [81]. Pectinesterase (PME) mainly catalyze the demethylation of pectin to form pectic acid and methanol [82]. The pectic acid can form pectin gel substances with Ca^2+^ and some other ions in bridging interaction, which strengthens the intercellular adhesion and enhances the robustness of plant tissue cell walls, thus improving the plant's resistance to low temperature stress. For example, when maize and Arabidopsis were subjected to environmental stress, their PMEs expressions were significantly altered, thereby promoting the plant resistance increased [83, 84]. In this study, the expression level of *StPME4759.3* showed a gradual increase as the treatment temperature decreased and reached the maximum at -2 °C. It increased the plant cell wall stiffness by catalyzing the formation of pectic acid from pectin, thus improving the plant's ability to resist low-temperature stress. However, there were some differences between its trend and the change in soluble sugar content, which might be due to the fact that the synthesis of soluble sugar was regulated by multiple genes and multiple metabolic pathways. However, the important role played by PMEs in plant response to low temperature stress was certain.

The genes of the calcium-dependent protein kinase family play an important role in plant development and growth and in response to stress. They act as calcium signal receptors and transmitters in the Ca^2+^-mediated signal pathway and transmit signals to downstream regulatory networks. Thereby regulating the development and growth of plants and the response to adversity stress [85]. The *StCDPK16* in this study mainly catalyzed the phosphorylation of apoplast Ca^2+^ into the cytoplasm. Its expression level gradually increased with the temperature decreased, and promoted the transfer of Ca^2+^ from outside the cell to the inside of the cell. By increasing the intracellular Ca^2+^ concentration, it can enhance the function of Ca^2+^ as a second messenger and it can also promote the formation of pectin gel under the bridging action of pectic acid and Ca^2+^, enhance the stability of the cell wall. This is consistent with the findings of Singh A and Dong H et al. [86, 87].

Phosphatidate phosphatases are a group of lipid phosphatases that catalyze the dephosphorylation of lipids containing phosphate groups to produce diacylglycerol. Diacylglycerol can be further metabolized to form triacylglycerol, which acts as a lipid signaling molecule and is involved in plant development and growth and stress. In this study, *StLPIN10369.5* mainly catalyzed the conversion of diacyl-sn-glycerol 3-phosphate to diacyl-sn-glycerol, ultimately formed triglycerides to improve plant resistance to low temperature stress. This was consistent with the results of studies in Arabidopsis and peanut [88, 89].

Pyrroline-5-carboxylic acid reductase is an important class of housekeeping proteins commonly found in prokaryotes and eukaryotes. It catalyzes the conversion of pyrroline-5-carboxylic acid to proline, the final step in proline synthesis [90]. In addition to acting as an osmoregulatory substance in the plant cytoplasm, proline accumulated in plants also plays an important role in stabilizing the structure of biomolecules, relieving ammonia toxicity and regulating cellular redox reactions. The increase of proline in plant tissues under low temperature conditions can improve the cold resistance of plants [91, 92]. In this study, *StproC27072* mainly catalyzed the formation of L-proline from L-1-pyrroline-5-carboxylic acid, and its expression showed an upward and then downward trend with decreasing temperature, but the content of proline gradually increased as the treatment temperature decreased, and reached the maximum at -2 °C. This might be because low-temperature freezing inhibited the activity of enzymes related to *StproC27072* expression and also inhibited the activity of enzymes related to proline degradation to a greater extent, thus resulting in a continued increase in the content of proline when the expression of *StproC27072* decreased.

## Conclusions

Full-length transcriptome sequencing is a fundamental resource to study of functional, structural, and comparative genomics that are almost none in potatoes and their relatives. In this paper, we sequenced full-length transcriptomes in potatoes, by using a combination of second generation and third generation sequencing. According to the previous results,

we have clarified that the potatoes respond to low-temperature stress through the following steps: 1. Low-temperature stress promoted the expression of *StLPIN10369.5* and *StCDPK16* in potato seedlings, and while produced diacyl-sn-glycerol, it also catalyzed the transfer of Ca^2+^ from extracellular to intracellular; 2. Diacyl-sn-glycerol and Ca^2+^ accumulated in a large amount in the cell, and they act as the second messenger to initiate the expression of related genes such as *StPME4759.3* and *StproC27072* in the cell in response to low-temperature stress; 3. *StPME4759.3*, activated by the second messenger in the cell, catalyzed pectin to form pectic acid; StproC27072 catalyzed L-1-pyrroline-5-carboxylic acid to form L-proline; 4. The intracellular accumulation of Ca^2+^ and pectic acid formed pectin gel through bridging action; diacyl-sn-glycerol was further metabolized to form triglycerides; 5. The massive formation of pectin gel increased the stability of the cell wall; the formed triglycerides increased the fluidity of the cell membrane; the increased Proline could not only regulate the osmotic pressure in the cell, but also stabilize the structure of biological macromolecules and regulate intracellular redox response, thereby improving the plant's ability to resist low temperature stress. These results suggested that *StLPIN10369.5* and *StCDPK16* may play a central coordinating role in response to low temperature stress in the potato. Our study provided a comprehensive transcriptome database for the response of potatoes to low temperature.

## Materials and methods

### Materials

The plant materials used in the present research were *Solanum tuberosum* L. cv*.* Favorita, which was obtained from Anhui Academy of Agriculture Sciences. The potato plants were grown in a growth chamber with a controlled environment of 22 ± 2 °C under a 16 h light/8 h dark photoperiod for 35 days. The growth potential of the same plants was homogenized for 7 days in a 20 °C degree artificial climate incubator. The test materials were divided into the control group and the treatment group. The control group was that the plants grown at 20 °C for 4 h (S 20, T1-T3). The treatment group was that the plants grown at 2 °C for 4 h (S 2, T10-T12) and at -2 °C for 4 h (S -2, T13-T14). The third fully expanded leaf was collected after the treatments, immediately frozen in liquid nitrogen and stored at − 80 °C. The RNA samples were extracted from potato leaves and stored at − 80 °C. All samples were sent to Biomarker Technologies for sequencing [93]. The samples were also used for qRT-PCR analysis.

### Methods

The statistical analysis of the data of MDA, proline and soluble sugar content in this manuscript used SPSS 13 software, and the mappings used Excel 2010.

### Determination of Malondialdehyde content

Determination of MDA content in potato plants by ultraviolet spectrophotometry [94], the specific measurement steps were as follows:

1. MDA extraction: 0.5 g potato leaves were chopped and placed them in a mortar, added 5 ml, 5% trichloroacetic acid and ground them into a homogenate, transfered all the homogenates into a centrifuge tube, centrifuged at 12,000 rpm at 4 °C for 10 min, and collected the supernatant;

2. Sample determination: Taken 2 ml of the supernatant and added 2 ml of 0.67% Thiobarbituric acid (TBA), mixed them in a water bath at 100 °C for 30 min, cooled down and centrifuged at 12,000 rpm for 4 min at 4 °C, and collected the supernatant, taken 3 ml of supernatant and measured its absorbance at 450 nm, 532 nm and 600 nm respectively.

## MDA content calculation: MDA (µmol/g) = [MDA concentration (µmol/L) × Extract volume (L)]/ Fresh leaf weight (g)

MDA concentration = 6.45 × (A532-A600) – 0.56 × A450.

A450: The absorbance value at 450 nm wavelength.

A532: The absorbance value at 532 nm wavelength.

A600: The absorbance value at 600 nm wavelength.

### Determination of Proline content

Proline content in potato plants was determined by the spectrophotometric method [94], the specific measurement steps were as follows:

1. Proline extraction: 0.5 g of potato leaves were taken, added 5 ml of 3% sulfosalicylic acid and grind. The grinding liquid was leached in a boiling water bath for 10 min and then cooled. The cooling liquid was centrifuged at 4 °C at 3000 rpm for 10 min. Taken 2 ml of supernatant liquid + 2 ml of glacial acetic acid (100%) + 3 ml acid ninhydrin (Acid ninhydrin configuration: taked 2.5 g of ninhydrin and added 60 ml, 100% glacial acetic acid and 40 ml, 6 mol/L phosphoric acid, dissolved by heating at 70 °C, cooled and stored at 4 °C in a brown reagent bottle), and heated them in a boiling water bath for 30 min. After cooling, added 5 ml of toluene, shaken well, let stand for stratification, and taken the supernatant.

2. Sample determination: Used blank as a control at 520 nm to measure the absorbance of the supernatant.

3. Content calculation: Proline content (µg/g) = [(C × Dilution times × Total amount of extract (ml)] / [Sample fresh weight (g) × Amount of extract (ml)]. C is the micrograms of proline found on the standard curve.

### Determination of Soluble sugar content

Determination of soluble sugar content in potato plants by Anthrone method [94], the specific measurement steps were as follows:

1. 0.1 g of potato leaves were taken, cut them into small pieces, and put them into a test tube; 2. Added 10 ml of distilled water to the test tube and put it in a boiling water bath for 4 consecutive extractions, and collected the extract; 3. After decolorizing the extract with activated carbon, filtered it with filter paper and dilute the filtrate to 100 ml, taken 0.2 ml filtrate and added 1.8 ml distilled water and mix well; 4. Mixed liquid anthrone ethyl acetate 0.5 ml + concentrated H_2_SO_4_ 5 ml, after fully shaking, put the test tube in a boiling water bath for 1 min and taken it out, measured the absorbance at 630 nm after natural cooling, and used the blank as a control.

5. Content calculation: Soluble sugar content = [(M_x_ × V × D) / (V_1_ × W × 10^3^) × 100] × 100%

M_x_: Soluble sugar calculated by standard equation (ug).

V: Total sample volume (ml).

V_1_: Sampling volume during measurement (ml).

D: Dilution times.

10^3^: The sample is converted from ‘mg’ to ‘ug’ multiples.

W: The sample fresh weight (g).

### RNA isolation and detection

Total RNA was isolated from the above samples using TRIzol reagent (Invitrogen, CA, USA) according to the manufacturer’s instructions, the amount of plant tissue for RNA extraction was 300 mg. RNA purity was checked using the NanoPhotometer® spectrophotometer (IMPLEN, CA, USA). RNA concentration was measured using Qubit® RNA Assay Kit in Qubit®2.0 Fluorometer (Life Technologies, CA, USA). RNA integrity was assessed using the RNA Nano 6000 Assay Kit and the Agilent Bioanalyzer 2100 system (Agilent Technologies, CA, USA).

### Illumina transcriptome library preparation and sequencing

A total of 1 μg RNA per sample was used as input material to generate sequencing libraries using the NEBNext UltraTM RNA Library Prep Kit for Illumina (NEB, USA) following the manufacturer’s recommendations, and index codes were added to attribute sequences to a specific sample. Briefly, mRNA was purified from total RNA using poly-T oligo-attached magnetic beads. Fragmentation was carried out using divalent cations under elevated temperature in the NEBNext First Strand Synthesis Reaction Buffer (5X). First strand cDNA was synthesized using random hexamer primers and MMuLV Reverse Transcriptase (RNase H-). Second strand cDNA synthesis was subsequently performed using DNA polymerase I and RNase H. Remaining overhangs were converted into blunt ends via exonuclease/polymerase activities. After adenylation of 3’ ends of DNA fragments, NEBNext Adaptors with hairpin loop structures were ligated to prepare for hybridization. In order to select cDNA fragments 200–250 bp in length, the library fragments were purified with the AMPure XP system (Beckman Coulter, Beverly, USA). Then 3 μl USER Enzyme (NEB, USA) was incubated with size-selected, adaptor-ligated cDNA at 37 °C for 15 min followed by 5 min at 95 °C. Then PCR was performed with Phusion High-Fidelity DNA polymerase, Universal PCR primers and Index (X) Primer. Finally, PCR products were purified (AMPure XP system) and library quality was assessed on the Agilent Bioanalyzer 2100 system. The clustering of index-coded samples was performed on a cBot Cluster Generation System using the TruSeq PE Cluster Kit v4-cBot-HS (Illumina) according to the manufacturer’s instructions. After cluster generation, the libraries were sequenced on an Illumina Hiseq X Ten platform, and paired-end reads were generated.

### PacBio Iso-Seq library preparation and sequencing

High-quality RNAs from control and low-temperature treated potato leaves were combined to obtain the PacBio Iso-seq libraries. At the same time, control-temperature /low-temperature potato leaves Iso-seq hybrid libraries were constructed by preparing multiple size fractionated cDNA and cells (2 cells for 1–2 kb, 1 cell for 2–3 kb, 1 cell for 3–6 kb). This approach avoids loading bias while obtaining a larger number of RNA sequences representative of gene expression profiles in control-temperature and low-temperature treated potato leaves.

The sequencing library was prepared according to the Iso-Seq protocol as described by Pacific Biosciences (P/N100-377–100-05 and P/N100-377–100-04). The SMARTer PCR cDNA Synthesis Kit was used to synthesize cDNA from the same RNA samples used for Illumina sequencing. After 23 cycles of PCR amplification, products were size selected using the BluePippin Size Selection System with the following bins for each sample: 1–2 kb, 2–3 kb and 3–10 kb. The amplified cDNA products were used to generate SMRTbell Template libraries according to the Iso-Seq protocol. Libraries were prepared for sequencing by annealing a sequencing primer and adding polymerase to the primer annealed template. The polymerase-bound template was bound to MagBeads and sequencing was performed on a PacBio RSII instrument. PacBio's sequencing principle is to use a polymerase enzyme to confine the replication of DNA to a tiny gap and to add fluorescent tracer markers to various bases. When the bases synthesize DNA strands, these fluorescent markers will emit different colors. According to the color of the flash, different bases can be identified.

### Illumina data analysis

Raw data (raw reads) in fastq format were first processed using in-house perl scripts. In this step, clean data (clean reads) were obtained by removing reads containing adapters, reads containing polyN and low-quality reads. These clean reads were then mapped to the reference genome sequence (PGSC_DM_v4.03) using STAR. Only reads with a perfect match or one mismatch were further analyzed and annotated based on the reference genome. Gene expression levels were estimated by FPKM (Fragments Per Kilobase per Million). Differential expression analysis between two conditions groups was performed using the DESeq R package (1.10.1) [95]. The resulting P values were adjusted using the Benjamini and Hochberg’s approach for controlling the false discovery rate [96]. Genes identified by DESeq with FDR ≤ 0.01 and FC ≥ 2 were defined as differentially expressed. K-means clustering was conducted based on Pearson correlation of gene expression profiles [97].

### PacBio data analysis

SMRT-Analysis software package v3.0 (https://github.com/ben-lerch/IsoSeq-3.0/blob/master/README.md) was used for Iso-Seq data analysis. First, reads of insertion (ROIs) were generated using the minimum filtering requirement of 0 or greater passes of the insert and a minimum read quality of 75. Then, full-length non-chimeric reads (FLNCs) containing the 5′ and 3′ adapters used in the library preparation as well as the poly (A) tail were identified.

### Identification of AS events, SSRs, CDSs and APA

The transcripts data was used to perform all-vs-all BLAST, and the BLAST alignments that met all criteria were considered as the products of the candidate AS events. The AS gap was larger than 100 bp and at least 100 bp away from the 3′/5′ end. SSRs within the transcriptome were identified by MISA (http://pgrc.ipk-gatersleben.de/misa/). TransDecoder (https://github.com/TransDecoder/TransDecoder/releases) was used to identify CDS regions within the transcript sequences. And the APA was identified by TARDIS pipeline [35].

### Functional annotation of genes

Genes were annotated by conducting blastx searches against public databases, including the NCBI non-redundant protein database (Nr), NCBI non-redundant nucleotide database (Nt), SwissProt, Protein Family (Pfam), Gene Ontology (GO), and the Kyoto Encyclopedia of Genes and Genomes (KEGG), with an E-value threshold of 10^−5^. GO enrichment analysis was performed using topGO with Fisher’s exact test, and the negative log10 transformed P-values were visualized using heatmaps as previously described [98, 99].

### Quantification of gene expression levels

In order to examine the expression of differential expression genes in potatoes, total RNAs of the samples were extracted using the Trizol reagent (Invitrogen) according to the manufacturer’s instructions. The DNase-treated RNA was reverse-transcribed using M-MLV reverse transcriptase. The ef1-α was used as an internal reference with primers synthesized by Sangon Biotech Co., Ltd (Additional file 10) [100]. The ABIStepOne PULS instrument was used for qRT-PCR experiments on cDNA samples from samples. Experiments were repeated 3 times. Relative gene expression levels were calculated by the 2^−ΔΔCT^ method. Reactions contained the following: 10 μl of 2xTransStar ® SybrGreen qPCR Master Mix, 2 μl of template cDNA, 0.4 μl of forward and Universal miRNA qPCR Primer, 0.4 μl of Passive Reference Dye and water to 20 μl. PCR amplification was carried out as follows: 50 °C for 2 min, 95 °C for 3 min, followed by 45 cycles of 95 °C for 5 s, 60 °C for 30 s.

## Supplementary Information

**Additional file 1:**

**Additional file 2.**

**Additional file 3.**

**Additional file 4.**

**Additional file 5.**

**Additional file 6.**

**Additional file 7.**

**Additional file 8.**

**Additional file 9.**

**Additional file 10.**

## Data Availability

The sequencing data of this study are available in the Sequence Read Archive (SRA) at the National Center for Biotechnology Information (NCBI) (accession number: PRJNA PRJNA748542 and PRJNA587793). The other supporting data are included as Supplemental Files.

## Abbreviations

MDA: Malondialdehyde
Pro: Proline
SGS: Second-generation sequencing technology
TGS: Third-generation sequencing
DEG: Differentially expressed gene
TF: Transcription factor
ROIs: Reads of insertion
CCS: Circular consensus
FLNC: Full-Length non-chimeric Read
ICE: Iterative isoform-clustering
CEC: Clustering for error correction
SMRT: Single-molecule real-time sequencing
NR: NCBI non-redundant proteins
NT: NCBI non-redundant nucleotide database
GO: Gene Ontology
KEGG: Kyoto Encyclopedia of Genes and Genomes
CC: Cellular component
MF: Molecular function
BP: Biological process
AS: Alternative splicing
SSR: Simple repetitive sequence
CDS: Coding sequence
APA: Alternative polyadenylation
PME: Pectinesterase
Ca^2^^+^: Calcium ion
