# Supplementary material for: Full-length transcriptome sequencing reveals the molecular mechanism of potato seedlings responding to low-temperature
Source: BMC Plant Biol. 2022 Mar 18;22:125. doi: 10.1186/s12870-022-03461-8 (PMC8932150; doi:10.1186/s12870-022-03461-8)
Supplement: Supplementary file 1 — Additional file 1: [file 12870_2022_3461_MOESM1_ESM.docx]

Table S1 Full-length transcriptome sequencing data statistics table

| **Sample Name** | **Clean data (GB)** | **cDNA size** | **SMRT Cells** | **Polymerase Reads** | **Post-Filter Polymerase Reads** | **Post-Filter Total Number of Subread Bases** | **Post-Filter Number of Subread** | **Post-Filter Subreads N50** | **Post-Filter Mean Subread length** |
| --- | --- | --- | --- | --- | --- | --- | --- | --- | --- |
| F01 | 8.27 | 1-2K | 2 | 300584 | 173407 | 3735625858 | 2296502 | 1606 | 1626 |
| F01 |  | 2-3K | 1 | 150292 | 107341 | 3012269439 | 1183048 | 2417 | 2546 |
| F01 |  | 3-6K | 1 | 150292 | 81540 | 1976407798 | 510570 | 3733 | 3870 |

Table S2 ROI data statistics table

| **Samples** | **cDNA size** | **Reads of Insert** | **Read Bases of Insert** | **Mean Read Length of Insert** | **Mean Read Quality of Insert** | **Mean Number of Passes** |
| --- | --- | --- | --- | --- | --- | --- |
| F01 | 1-2K | 122,575 | 252,991,528 | 2,063 | 0.92 | 13.00 |
| F01 | 2-3K | 77,994 | 234,144,767 | 3,002 | 0.93 | 10.00 |
| F01 | 3-6K | 44,918 | 181,759,393 | 4,046 | 0.89 | 6.00 |
| F01 | All | 245,487 | 668,895,688 | 2,724 | 0.92 | 10.00 |

Table S3 Full-length sequence data statistics table

| **Samples** | **cDNA Size** | **Reads of Insert** | **Number of five prime reads** | **Number of three prime reads** | **Number of poly-A reads** | **Number of filtered short reads** | **Number of non-full-length reads** | **Number of full-length reads** | **Number of full-length non-chimeric reads** | **Average full-length non-chimeric read length** | **Full-Length Percentage (FL%)** | **Artificial Concatemers（%）** |
| --- | --- | --- | --- | --- | --- | --- | --- | --- | --- | --- | --- | --- |
| F01 | 1-2K | 122,575 | 71,915 | 79,900 | 77,397 | 15,348 | 45,692 | 61,535 | 60,911 | 1,293 | 50.20% | 1.01% |
| F01 | 2-3K | 77,994 | 47,092 | 50,428 | 49,318 | 7,201 | 30,843 | 39,950 | 39,755 | 2,099 | 51.22% | 0.49% |
| F01 | 3-6K | 44,918 | 24,944 | 26,640 | 26,561 | 1,594 | 22,669 | 20,655 | 20,637 | 3,464 | 45.98% | 0.09% |
| F01 | All | 245,487 | 143,951 | 156,968 | 153,276 | 24,143 | 99,204 | 122,140 | 121,303 | 1,926 | 49.75% | 0.69% |

Table S4 ICE clustering result statistics table

| **Samples** | **Size** | **Number of consensus isoforms** | **Average consensus isoforms read length** | **Number of polished high-quality isoforms** | **Number of polished low-quality isoforms** | **Percent of polished high-quality isoforms(%)** |
| --- | --- | --- | --- | --- | --- | --- |
| F01 | 0to1kb | 3,919 | 909 | 3,428 | 491 | 87.47% |
| F01 | 1to2kb | 25,737 | 1,506 | 21,618 | 4,119 | 84.00% |
| F01 | 2to3kb | 11,067 | 2,294 | 8,801 | 2,266 | 79.52% |
| F01 | 3to6kb | 11,186 | 3,535 | 7,162 | 4,024 | 64.03% |
| F01 | above6kb | 571 | 9,870 | 7 | 564 | 1.23% |
| F01 | All | 52,480 | 2,151 | 41,016 | 11,464 | 78.16% |
