# Supplementary material for: Full-length transcriptome sequencing reveals the molecular mechanism of potato seedlings responding to low-temperature
Source: BMC Plant Biol. 2022 Mar 18;22:125. doi: 10.1186/s12870-022-03461-8 (PMC8932150; doi:10.1186/s12870-022-03461-8)
Supplement: Supplementary file 2 — Additional file 2. [file 12870_2022_3461_MOESM2_ESM.docx]

Table S5 Statistics table of the sequence comparison results of transcriptome sequencing data and reference genome

| **Sample** | **Total Reads** | **Uniquely mapped reads %** | **% of reads mapped to multiple loci** | **% of reads mapped to too many loci** |
| --- | --- | --- | --- | --- |
| T01 | 28270361 | 81.05% | 5.52% | 0.08% |
| T02 | 27318299 | 82.24% | 5.87% | 0.07% |
| T03 | 32928931 | 81.98% | 5.81% | 0.09% |
| T04 | 24525892 | 80.98% | 6.62% | 0.32% |
| T05 | 24275418 | 78.70% | 7.13% | 0.42% |
| T06 | 26140364 | 82.85% | 4.89% | 0.05% |
| T07 | 24759490 | 82.24% | 5.33% | 0.12% |
| T08 | 24210388 | 81.82% | 4.87% | 0.07% |
| T09 | 25615720 | 79.14% | 6.10% | 0.20% |

Note: Total Reads is the number of Clean Reads; Uniquely mapped reads %: the number of Reads that are compared to the unique position of the reference genome and the percentage in Clean Reads;% of reads mapped to multiple loci: the number of Reads that are compared to multiple positions in the reference genome And the percentage in Clean Reads;% of reads mapped to too many loci: The number of Reads that are compared to many positions in the reference genome and the percentage in Clean Reads
