# Supplementary material for: Full-length transcriptome sequencing reveals the molecular mechanism of potato seedlings responding to low-temperature
Source: BMC Plant Biol. 2022 Mar 18;22:125. doi: 10.1186/s12870-022-03461-8 (PMC8932150; doi:10.1186/s12870-022-03461-8)
Supplement: Supplementary file 10 — Additional file 10. [file 12870_2022_3461_MOESM10_ESM.docx]

**Supplementary table. Primers for qRT-PCR**

| **Homolog** | **Forward primer sequence (5’ to 3’)** | **Reverse primer sequence (5’ to 3’)** |
| --- | --- | --- |
| *StPME4759.3* | GGTAGAGGCTTCATTGCCAGAG | GTTGGAGTGAGCATAAAGGGTGT |
| *StCDPK16* | GTCGCAAGCCATGGTCTAAC | CTAACGCCCGTAAAGCAAAC |
| *StLPIN10369.5* | TTCTAGTGAGCGTGGATGGC | CGTCGCAAGTGTCTCCTGAT |
| *StproC27072* | GTGTCAGTTGCTGCTGGTGTT | CCATCTTCTGTTGTTGCCTTTT |
| *ef1-α* | ATTGGAAACGGATATGCTCCA | TCCTTACCTGAACGCCTGTCA |
